# Clinical profile of congenital heart diseases detected in a tertiary hospital in China: a retrospective analysis

**DOI:** 10.3389/fcvm.2023.1131383

**Published:** 2023-09-08

**Authors:** Linhong Song, Yi Wang, Hui Wang, Gang Wang, Ning Ma, Qiang Meng, Kunao Zhu, Siqi Hu, Gengxu Zhou, Zhichun Feng

**Affiliations:** ^1^ The Second School of Clinical Medicine, Southern Medical University, Guangzhou, China; ^2^ Department of Pediatric Cardiac Surgery, Faculty of Pediatrics, The Seventh Medical Center of Chinese PLA General Hospital, Beijing, China; ^3^ Institute of Pediatrics, The Seventh Medical Center of Chinese PLA General Hospital, Beijing, China; ^4^ National Engineering Laboratory for Birth Defects Prevention and Control of Key Technology, Beijing, China; ^5^ Beijing Key Laboratory of Pediatric Organ Failure, Beijing, China

**Keywords:** congenital heart defects, clinical presentation, cardiovascular complications, pediatrics, pediatric cardiology

## Abstract

**Background:**

Congenital heart diseases (CHDs) are conditions that involve structural problems to the heart's structure existing at birth, with an incidence of approximately 8 per 1,000 live births globally. CHD is one of the leading causes of maternal, fetal, and neonatal morbidity and mortality worldwide. The present study sought to examine the clinical profiles of CHD patients and provide important implications for therapeutic interventions.

**Methods:**

This was a retrospective, observational, cohort study. The medical records of all CHDs patients aged between 0 and 18 years were collected from July 1, 2021 to June 30, 2022. Clinical profiles and demographic data were collected from cardiology and pediatric department registers for analysis.

**Results:**

Of the 265 children with CHDs, 201 were diagnosed with acyanotic CHD (ACHD), while 64 children had cyanotic CHD (CCHD). Based on the eleventh revision of the International Classification of Diseases (ICD-11), “congenital anomaly of a ventricle or the ventricular septum” was the most common CHD. The most common symptom was failure to thrive, accounting for 18.5% of all CHD cases. The most frequent symptom in ACHD was murmur (93.53%) and sweating (80.60%), whereas the most common symptom in CCHD was sweating (95.31%) and cyanosis (84.38%).

**Conclusions:**

This study retrospectively analyzed CHD clinical characteristics from children receiving care at the seventh center, which forms a proper basis for appropriate clinical treatments and further studies.

## 1. Introduction

Congenital heart diseases (CHDs) are the most common type of congenital birth defect and the leading cause of infant morbidity and mortality worldwide. The incidence of CHD in different studies varies from approximately 4/1,000 to 50/1,000 live births (1). Recently, a meta-analysis demonstrated that the prevalence (1970–2017) of CHD diagnoses first made in childhood was 1.384/1,000 (2). CHD is often classified into two types based on the pathophysiology and the affected heart structure: acyanotic CHD (ACHD) and cyanotic CHD (CCHD) (3). CCHD involves heart defects in which the blood delivered to the body contains less-than-normal amounts of oxygen. ACHDs are cardiac malformations that affect normal blood flow.

Until recently, the exact etiology of the majority of CHDs is unknown. To explain the pathogenesis of CHDs, several theories have been put forward. In 1968, Nora introduced the multifactorial inheritance hypothesis, which suggested that CHD results from genetic-environmental interaction (4). CHDs may be related to gross chromosomal or genetic aberrations, smoking or drinking, and illnesses in the mother during pregnancy. In recent times, the utilization of comprehensive techniques such as whole-exome sequencing (WES) and whole-genome sequencing (WGS), along with advanced bioinformatic tools and extensive, aggregated, population-based sequencing datasets, has facilitated the recognition of detrimental missense and loss-of-function (LOF) variants—collectively referred to as damaging variants. This approach also extends to the detection of small insertions or deletions, copy number variants (CNVs), and structural variants (5). These approaches identify damaging coding variants in definitive and candidate genes for CHD in 45% of patients with CHD (6–9). So far, more 400 genes have been implicated in the development and progression of CHD, encompassing transcription factors, cell signaling molecules, structural proteins, chromatin modifiers and cilia related proteins that are important for heart development (5, 10). CHD symptoms may present at birth or may appear later in life. Signs and symptoms of CHDs vary from mild to severe depending on the severity and type of the heart defect. Some heart defects might have little or no symptoms while others might have serious symptoms, including blueish skin, lips, or nails (cyanosis), hypersomnia, troubled breathing or fast breathing, poor circulation, getting unusually tired or breathing difficulty when exercising, heart murmur (a whooshing or swishing sound made by turbulent blood flow through the heart valves), and pounding heartbeat or weak pulse.

Psychological stress symptoms such as depression and anxiety are common among patients with CHDs. In addition, families with CHD patients are faced with financial hardships (11–13). Therefore, CHD is considered to be one of the leading diseases with the highest burden.

CHDs can further be categorized according to the degree of severity: mild CHDs with relatively little need for healthcare and severe or highly complex CHDs, which demand significantly greater expertise (14). Delayed diagnosis and intervention may promote relative complications such as severe cyanosis, heart failure, and other long-term sequelae in children (15–17). Recently, advances in cardiovascular diagnostics and medical treatments have critically improved the survival rate and the quality of life of patients with CHDs (18, 19). Thus, understanding the clinical profile of CHDs is important for better management of these diseases (20, 21).

CHD is a serious threat to the health of children and is one of the top five causes of death in children under 5 years of age in China. With rapid economic development in China, the mortality rate of CHD children under 5 years of age has decreased significantly in both rural and urban areas (22, 23). While the mortality rate of surgical treatment of simple CHD lesions is relatively lower in China than in European countries, the mortality rate of complex CHD lesions is significantly higher in China than in European countries (23). Regional disparities in health give rise to a significant future disease burden (24). Thus, data on the clinical symptoms, severity, urban-rural disparity and phenotypes of CHDs in China is urgently needed.

Hence, the current study was conducted to explore the clinical profiles and manifestations of CHD among patients receiving medical care at the Seventh Medical Center of the PLA General Hospital, which is a tertiary care center for patients from northern China, with a well-established pediatric cardiac surgery department. We established a clinical database to conduct further analysis of clinical profiles of CHDs in patients aged 0–18 years, which is of great significance in understanding the types and trends in CHDs in northern China. Findings from this study may help design more targeted treatment protocols and prevention measures.

## 2. Materials and methods

### 2.1. Study design and participants

This is a retrospective review of prospectively collected data. The study was performed at the Seventh Medical Center of the PLA General Hospital. Patients were identified by trained cardiologists in our center. All patients were enrolled between July 1, 2021 and June 30, 2022. A total of 265 patients with CHD aged 0–18 years were included in this study. This study was approved by the Ethics Committee of the Seventh Medical Center of PLA General Hospital (2022-195).

### 2.2. Data collection

Clinical data were collected from the electronic medical record of the Seventh Medical Center of the PLA General Hospital. Clinical profiles and demographic data were obtained from cardiology and pediatric department registers.

### 2.3. Statistical analysis

Descriptive statistics, including age, region, International Classification of Diseases (ICD) diagnostic codes, and symptoms, were described as frequencies and percentages using GraphPad Prism version 7 for Windows (GraphPad, San Diego, CA). The chi-squared test was used to infer any differences between categorical variables. *P*-values of less than 0.05 were considered statistically significant. All statistical analyses were carried out using Statistical Package for the Social Sciences (SPSS) software version 20 (IBM Corp.; Armonk, NY, USA).

## 3. Results

### 3.1. Patient characteristics

**Figure 1:**
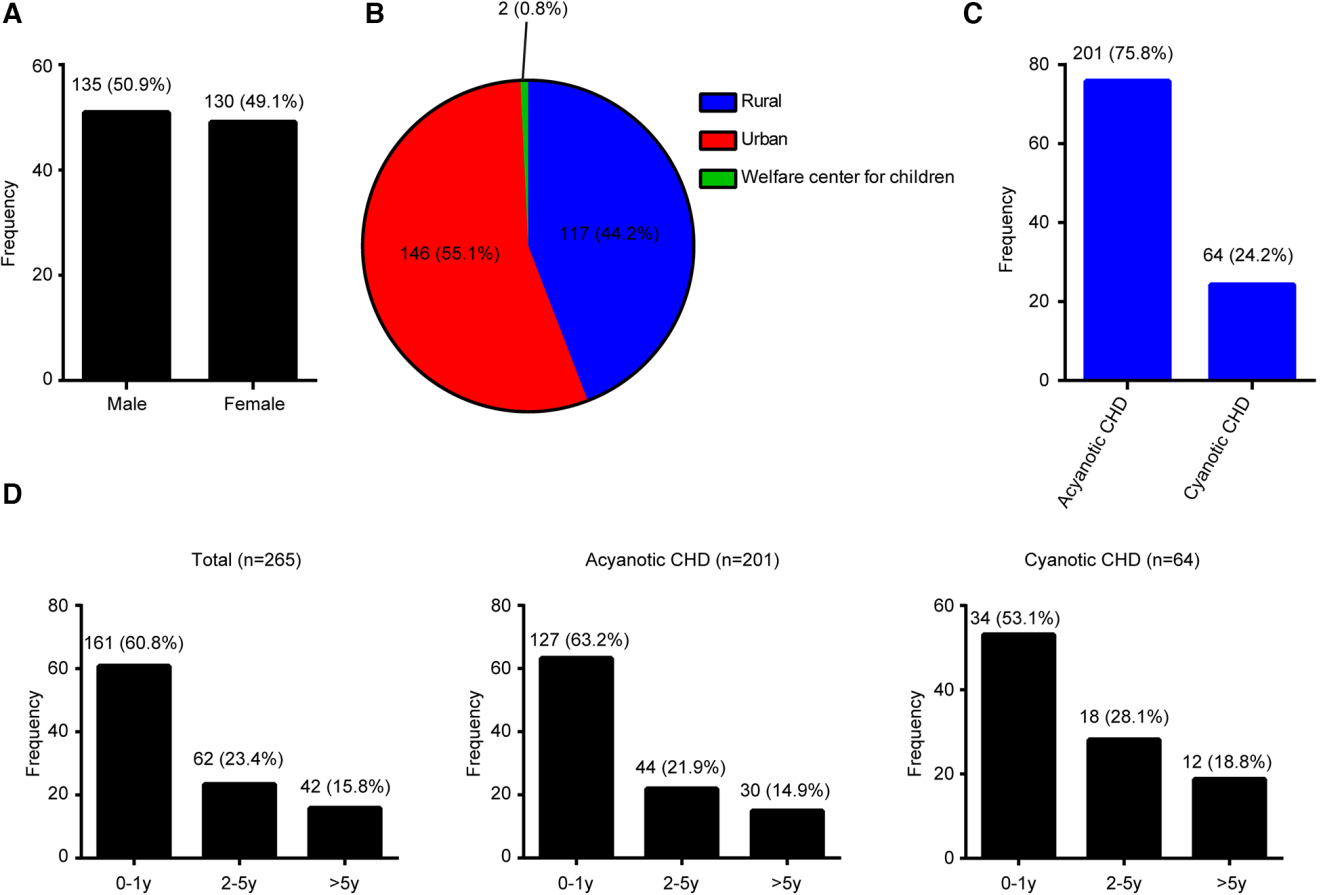
Disease distribution proportion in congenital heart disease patients. (**A**) Gender. (**B**) Location. (**C**) Type. (**D**) Age.

A total of 265 patients with CHD were enrolled, including 135 (50.9%) males and 130 (49.1%) females (Figure 1A), with a male-to-female ratio of 1.04:1. Of the 265 patients, 146 (55.1%) were from urban areas, 146 (44.2%) were from rural areas, and 2 (0.8%) were from Welfare center for children (Figure 1B). A total of 201 (75.8%) children were diagnosed with ACHD, while 64 (24.2%) children had CCHD (Figure 1C).

The age distribution of CHDs is shown in Figure 1D. The majority (60.8%, 161/265) of patients with CHDs were <1 year old, 23.4% (62/265) of patients were 1–5 years old, and 15.8% (42/265) were >5 years old. ACHD age distribution is shown in Figure 1D. About 63.2% (127/265) of patients were in the <1 year age group, 21.9% (44/265) were in the 1–5 years age group, and 14.9% (30/265) were in the >5 years age group. CCHD age distribution is shown in Figure 1D. Approximately 53.1% (34/265) of patients were in the <1 year age group, 28.1% (18/265) were in the 1–5 years age group, and 18.8% (12/265) were in the >5 years age group.

### 3.2. CHD profile

Based on the eleventh revision of the International Classification of Diseases (ICD-11), “congenital anomaly of a ventricle or the ventricular septum” constituted 124 cases, accounting for 46.80% of all CHD cases, followed by “congenital anomaly of a ventriculoarterial valve or adjacent regions” with 33 cases, accounting for 12.5% of all CHD cases, and “congenital anomaly of atrial septum” with 32 cases, accounting for 12.1% of all CHD cases. Other common types of CHD observed in CHD children at our hospital are listed in Table 1.

**Table 1:** Clinical spectrum of congenital heart diseases of study participants.

Disease	Code	Number
Congenital anomaly of a ventricle or the ventricular septum	LA88	124
Congenital anomaly of a ventriculoarterial valve or adjacent regions	LA8A	33
Congenital anomaly of the atrial septum	LA8E	32
Congenital anomaly of an atrioventricular valve or atrioventricular septum	LA87	21
Congenital anomaly of great arteries including arterial duct	LA8B	18
Congenital anomaly of an atrioventricular or ventriculoarterial connection	LA85	15
Congenital anomaly of mediastinal vein	LA86	15
Functionally univentricular heart	LA89	3
Conditions with disorders of intellectual development as a relevant clinical feature	LD90	3
Structural developmental anomalies of the peripheral vascular system	LA90	1

The prevalence of specific CHD types is prevented in Table 2. Our data share some variance with other studies (Table 2). Of all CHD types, ventricular septal defect (VSD) was the most common congenital cardiac anomaly in children (21, 25–30), accounting for 17.4% of all CHD cases in our study.

**Table 2:** Comparative study of lesions with other studies.

Type		Current study	(19)	(20)	(21)	(22)	(23)	(24)	(25)	(10)
Simple lesions	VSD	17.4	31.4	29.9	27.8	17.9	21.5	18.6	–	31.0
	ASD	7.2	2.0	25.4	20.3	8.9	9.3	18.8	18.9	23.0
	PDA	8.7	5.9	6.0	21.2	12.2	8.6	5.6	9.0	11.0
	VSD + ASD	10.2	–	3.7	–	2.4	–	–	–	3.0
	VSD + ASD + PDA	5.3	–	–	–	–	–	–	–	–
	VSD + PDA	1.5	–	–	2.66	8.1	–	–	–	–
	ASD + PDA	4.2	–	–	–	–	–	–	–	–
	PS	4.5	5.9	6.7	6.5	–	3.1	5.1	4.5	–
Complex lesions										
	COA	5.3	–	–	0.1	1.6	1.0	1.0	1.5	1.0
	TGA	2.3	2.0	5.2	–	8.1	4.9	3.4	2.0	4.0
	DORV	1.5	–	–	1.0	–	–	3.5	1.5	2.0
	TAPVR	5.7	–	–	–	–	–	0.7	–	1.0
	AS	1.9	–	–	0.7	–	1.9	0.9	1.5	1.0
	SV	1.1	–	–	–	–	–	–	–	2.0
	AS + COA	0.4	–	–	–	–	–	–	–	1.0
	TOF	3.8	25.5	11.2	5.5	24.4	24.4	13.4	8.0	11.5
	TA	1.1	–	–	0.1	–	–	1.8	0.5	0.5
	Ebstein anomaly	1.1	–	–	–	–	–	0.4	–	2.5
	IAA	0.8	–	–	–	–	–	–	–	0.5
	CECD	0.8	–	–	–	–	–	–	–	–
	PMV	2.3	–	–	–	–	–	–	–	–
	PA	4.9	–	–	–	–	–	0.3	–	–
	TR	0.4	–	–	–	–	–	–	–	–
	PI	0.4	–	–	–	–	–	–	–	–
	ccTGA	0.8	–	–	–	–	–	–	–	–
	MV	2.3	–	–	–	–	–	–	–	–
	MVS	0.8	–	–	–	–	–	–	–	–
	AI	0.4	–	–	–	–	–	–	–	–
	PAS	0.8	–	–	–	–	–	–	–	–
	AOPA	0.4	–	–	–	–	–	–	–	–
	CARP + PFO	0.4	–	–	–	–	–	–	–	–
	TS	0.4	–	–	–	–	–	–	–	–
	CAF + VSD + ASD + PDA + PH + ARSA + RAA	0.4	–	–	–	–	–	–	–	–
	SV + CA + PS + TGA	0.8	–	–	–	–	–	–	–	–
	SV + DORV + TAPVR + PS + CT	0.4	–	–	–	–	–	–	–	–

Information on the types, age distribution, and regional distribution of CHD is shown in Table 3. Out of 265 cases of CHD, 156 (58.9%) cases were simple lesions and 109 (41.1%) were complex lesions. About 22.3% of cases from rural areas were diagnosed as complex lesions after the first year of life, which was higher than those in urban areas (8.6%). This finding was statistically significant (*P* < 0.05). This may be because convenient and high-level medical conditions in urban areas enabled early detection of CHD.

**Table 3:** Types, age distribution, and regional distribution of congenital heart disease.

Type of CHD		Urban (117)					Rural (148)		
		Age range (years)					Age range (years)		
Simple lesions		<1	1–5	>5	Total	<1	1–5	>5	Total
	VSD	10	10	5	25	10	8	3	21
	ASD	2	2	3	7	2	6	4	12
	PDA	7	4	1	12	6	2	3	11
	VSD + ASD	9	0	0	9	18	0	0	18
	VSD + ASD + PDA	7	0	0	7	6	1	0	7
	VSD + PDA	1	0	0	1	2	1	0	3
	ASD + PDA	6	1	0	7	2	2	0	4
	PS	6	1	0	7	1	3	1	5
Sub-total (%)		48 (41.0)	18 (15.4)	9 (7.7)	75 (64.10)	47 (31.8)	23 (15.5)	11 (7.4)	81 (54.7)
Complex lesions									
	COA	4	0	0	4	9	0	1	10
	TGA	1	0	0	1	2	1	2	5
	DORV	2	0	0	2	0	1	1	2
	TAPVR	6	2	0	8	6	1	0	7
	AS	3	1	0	4	1	0	0	1
	SV	0	0	3	3	0	0	0	0
	AS + COA	0	0	0	0	0	0	1	1
	TOF	6	0	0	6	3	0	1	4
	TA	0	1	0	1	1	0	1	2
	Ebstein anomaly	0	0	0	0	1	1	1	3
	IAA	0	0	0	0	1	1	0	2
	CECD	1	0	0	1	1	0	0	1
	PMV	3	0	0	3	3	0	0	3
	PA	0	0	0	0	4	5	4	13
	TR	0	0	1	1	0	0	0	0
	PI	0	0	0	0	0	0	1	1
	ccTGA	0	0	0	0	0	2	0	2
	MV	2	0	0	2	1	2	1	4
	MVS	1	0	0	1	0	0	1	1
	AI				0	1	0	0	1
	PAS	0	1	0	1	0	1	0	1
	AOPA	1	0	0	1	0	0	0	0
	CARP + PFO	1	0	0	1	0	0	0	0
	TS	0	0	0	0	0	0	1	1
	CAF + VSD + ASD + PDA + PH + ARSA + RAA	1	0	0	1	0	0	0	0
	SV + CA + PS + TGA	0	0	1	1	0	1	0	1
	SV + DORV + TAPVR + PS + CT	0	0	0	0	0	1	0	1
Sub-total		32 (27.4)	5 (4.3)	5 (4.3)	42 (35.9)	34 (23.0)	17 (11.5)	16 (10.8)	67 (45.3)
Total					117				148

### 3.3. Clinical presentation of CHDs

**Figure 2:**
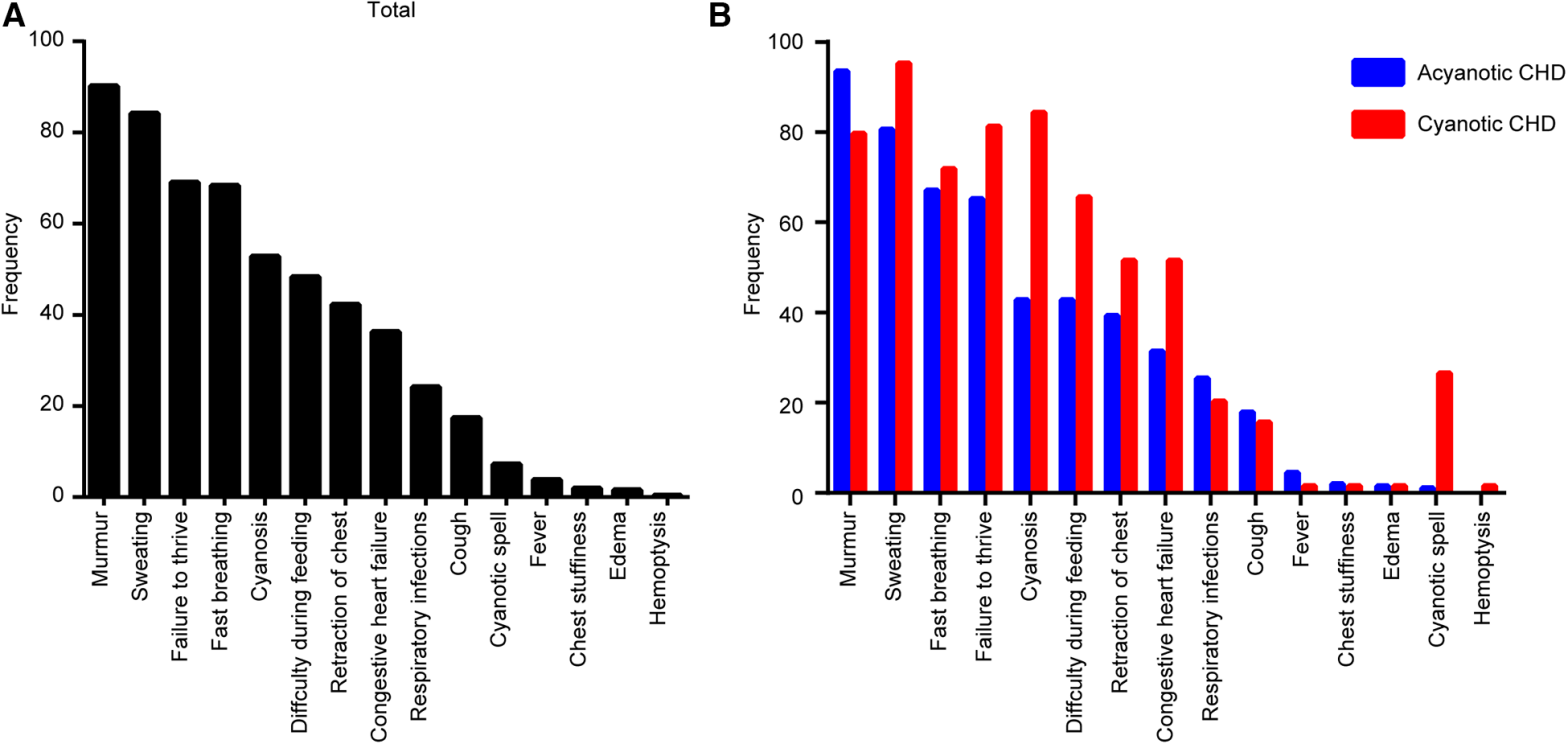
Clinical presentations associated with congenital heart disease (CHD) types in diagnosed children. (**A**) Total CHD. (**B**) Each type of CHD.

The symptoms of CHDs were analyzed and the most common symptoms were murmur (239/265, 90.2%), followed by sweating (223/265, 84.2%), failure to thrive (183/265, 69.1%), fast breathing (181/265, 68.3%), cyanosis (140/265, 52.8%), difficulty during feeding (128/265, 48.3%), retraction of the chest (112/265, 42.3%), congestive heart failure (96/265, 36.2%), respiratory infections (64/265, 24.2%), cough (46/265, 17.4%), cyanotic spell (19/265, 7.2%), fever (10/265, 3.8%), chest stuffiness (5/265, 1.89%), edema (4/265, 1.51%), and hemoptysis (1/265, 0.4%) (Figure 2A).

In the ACHD group, the most common symptoms included murmur (188/201, 93.5%), sweating (162/201, 80.6%), and fast breathing (135/201, 67.2%). In the CCHD group, the most common symptoms included sweating (61/64, 95.3%), cyanosis (54/64, 84.4%), and failure to thrive (52/64, 81.3%) (Figure 2B). Clinical symptoms of CHD are summarized in Table 4.

**Table 4:** Symptoms of each CHD.

Symptoms	CHD (*n* = 265)	ACHD (*n* = 201)	CCHD (*n* = 64)
Sweating	223	162	61
Cyanosis	140	86	54
Failure to thrive	183	131	52
Murmur	239	188	51
Fast breathing	181	135	46
Difficulty during feeding	128	86	42
Retraction of chest	112	79	33
Congestive heart failure	96	63	33
Cyanotic spell	19	2	17
Respiratory infections	64	51	13
Cough	46	36	10
Fever	10	9	1
Chest stuffiness	5	4	1
Edema	4	3	1
Hemoptysis	1	0	1

Clinical symptoms of CHD in our study and other studies are summarized in Table 5. Murmur was the most common symptom in the present study, which is consistent with the reported 49.7% and 81.4% in other studies (31, 32). Failure to thrive, fast breathing, and difficulty during feeding were the next common symptoms in other studies (21, 26, 31, 32).

**Table 5:** Comparative study of symptoms with other studies.

Symptoms	Current study (%)	(10)	(25)	(26)	(20)
Murmur	90.2	–	49.7	81.4	–
Sweating	84.2	–	–	40.9	–
Failure to thrive	69.1	55	17.9	61.4	48.5
Fast breathing	68.3	38	49.7	–	80
Cyanosis	52.8	23.5	41.3	3.8	31
Difficulty during feeding	48.3	34	40.2	38.6	58
Retraction of chest	42.3	45	–	5	–
Congestive heart failure	36.2	13	–	–	19.4
Cyanotic spell	7.2	9	–	–	–
Respiratory infections	24.2	–	21.9	–	23
Cough	17.4	38	17.9	50.5	53
Fever	3.8	44	–	–	41
chest stuffiness	1.9	–	–	–	–
Edema	1.5	55	–	7.3	–
Hemoptysis	0.4	–	–	–	–

## 4. Discussion

A total of 265 patients with CHD were enrolled in this study, it showed that the majority of children with CHDs were below one-year-old, which is consistent with findings from previous studies (21, 33). The ratio between the number of males and females was approximately the same (1.04:1), which is comparable with that in previous reports (31, 34). However, this ratio was 2:1 in some studies (21, 35, 36). “Congenital anomaly of a ventricle or the ventricular septum” was the most common CHD according to ICD-11. Fast breathing and cyanosis were the most common symptoms in ACHD and CCHD, respectively.

Approximately 22.3% of patients hailing from rural regions presented with complex lesions during the initial year of their lives. This percentage significantly surpassed the corresponding figure for patients residing in urban localities, which stood at 8.6%. This data underscores the burden experienced by individuals living in rural areas with undetected complex CHD lesions. This suggests that restricted access to healthcare, coupled with insufficient resources and referral systems, likely contributed to the missed diagnosis of these lesions. Optimization of health care resource allocation in poor rural areas and low-income households to facilitate early screening is an urgent policy priority in reducing the rate of missed diagnosis of complex CHD lesions.

According to Mitchell et al. (37), CHD is a gross structural abnormality of the heart or intrathoracic great vessels that is actually or potentially of functional significance. CHD may be identified at virtually any age. Certain symptoms and signs generally appear in neonates while others are rarely diagnosed during infancy (38).

The clinical symptoms of CHD can vary by the type and severity of the defect. Based on the data obtained in our study and other studies published from 2014 to 2022 (21, 26, 31, 32), CHD symptoms include murmur, failure to thrive, fast breathing, and difficulty during feeding during infancy and early childhood, with murmur being the most common symptom in our study and some previous studies (31, 32). However, some studies reported breathing difficulty as the most common symptom (26, 31).

CHD is the leading cause of birth defect-related mortality, accounting for approximately 40% of all deaths in children with birth defects worldwide (39–41). Prenatal screening by fetal echocardiography and postnatal detection by pulse oximetry combined with clinical assessment are the most common methods for CHD screening, which enables early detection of CHD lesions and proper implementation of suitable treatments (18, 42–44).

With improvements in people's living standards, the selection of elective surgery is becoming more scientific and rational. In addition, patients' guardians can assist doctors in decision-making, based on a more scientific basis. CHD is one of the top five causes of death in children under 5 years of age in China. Based on China's large population, the burden of CHD will be formidable in terms of healthcare services and economic costs in the future. Thus, future research should primarily focus on improving the treatment of complex CHD lesions in China.

## 5. Strength and limitations

To the best of our knowledge, this is the first study to assess clinical profiles of CHDs in China with considerable sample size. Nevertheless, it is a single-center, hospital-based study and not a community-based study. This study is a retrospective study and may contain some incomplete or missing data. Furthermore, this study was not designed to investigate the potential risk factors as predictors of CHDs.

## 6. Conclusions

In summary, this study retrospectively analyzed the clinical characteristics of children with CHD diagnosed at a tertiary center in China. Approximately 75.8% of subjects were diagnosed with ACHD and 24.2% were diagnosed with CCHD. “Congenital anomaly of a ventricle or the ventricular septum”, “congenital anomaly of a ventriculoarterial valve or adjacent regions”, and “congenital anomaly of atrial septum” were the top three diseases based on ICD-11. VSD was the most common congenital cardiac anomaly and murmur was the most common symptom in children with CHD. This is one of the first studies in the pediatric age group in northern China and may help to better understand the clinical characteristics and prevalence of CHDs in this region. Moreover, our data may assist in formulating policies for better management of CHDs.

## Data Availability

The original contributions presented in the study are included in the article/Supplementary Material, further inquiries can be directed to the corresponding authors.
